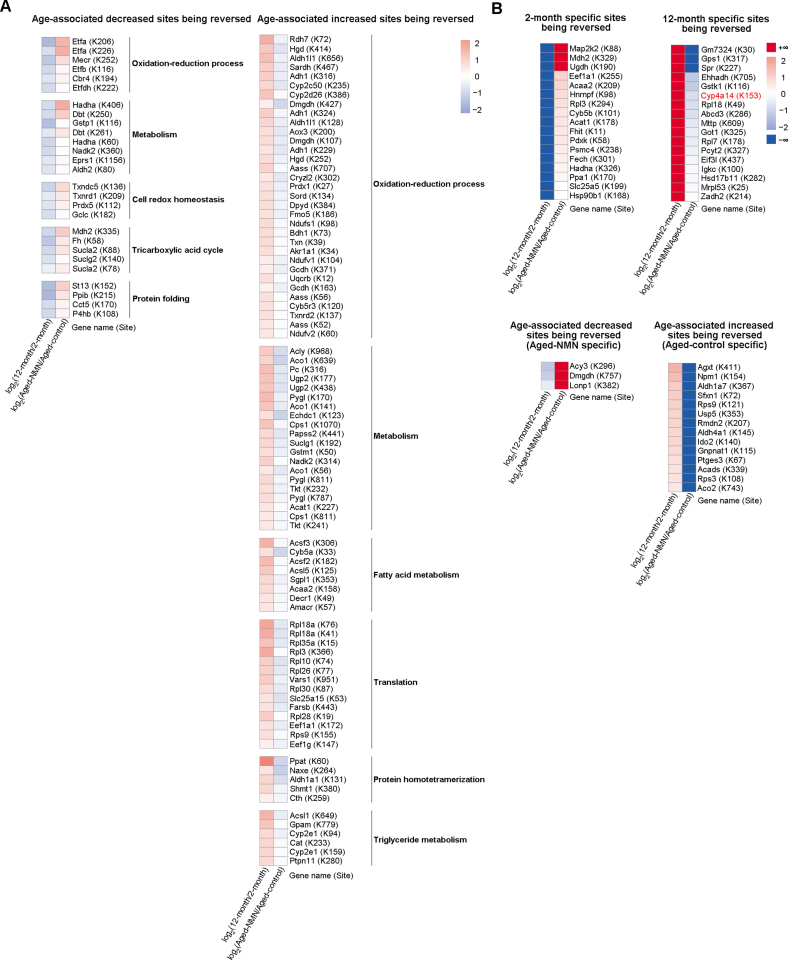# Quantitative Acetylomics Reveals Dynamics of Protein Lysine Acetylation in Mouse Livers During Aging and Upon the Treatment of Nicotinamide Mononucleotide

**DOI:** 10.1016/j.mcpro.2022.100276

**Published:** 2022-08-03

**Authors:** Jingshu Li, Ye Cao, Kongyan Niu, Jiaqian Qiu, Han Wang, Yingnan You, Dean Li, Yu Luo, Zhengjiang Zhu, Yaoyang Zhang, Nan Liu

**Affiliations:** 1 Interdisciplinary Research Center on Biology and Chemistry, Shanghai Institute of Organic Chemistry, Chinese Academy of Sciences, Shanghai, China; 2 University of Chinese Academy of Sciences, Beijing, China; 3 Abiochem Biotechnology, Shanghai, China

**Keywords:** Ac, acetylation, ACN, acetonitrile, DAVID, Database for Annotation, Visualization and Integrated Discovery, EDTA, ethylenediaminetetraacetic acid, FA, formic acid, GO, Gene Ontology, K, lysine, KEGG, Kyoto Encyclopedia of Genes and Genomes, LC-MS/MS, liquid chromatography with tandem mass spectrometry, MeOH, methanol, NAD^+^, nicotinamide adenine dinucleotide, NAM, nicotinamide, NaCl, sodium chloride, NH_4_OH, ammonium hydroxide, NH_4_OAc, ammonium acetate, NMN, beta-nicotinamide mononucleotide, PTM, post-translational modification, Tau, Microtubule-associated protein tau, TFA, trifluoroacetic acid, Tris-HCl, Tris-hydrochloride

## Abstract

Lysine acetylation is a reversible and dynamic post-translational modification that plays vital roles in regulating multiple cellular processes including aging. However, acetylome-wide analysis in the aging process remains poorly studied in mammalian tissues. Nicotinamide adenine dinucleotide (NAD^+^), a hub metabolite, benefits health span at least in part due to the activation of Sirtuins, a family of NAD^+^-consuming deacetylases, indicating changes in acetylome. Here, we combine two antibodies for the enrichment of acetylated peptides and perform label-free quantitative acetylomic analysis of mouse livers during natural aging and upon the treatment of beta-nicotinamide mononucleotide (NMN), a NAD^+^ booster. Our study describes previously unknown acetylation sites and reveals the acetylome-wide dynamics with age as well as upon the treatment of NMN. We discover protein acetylation events as potential aging biomarkers. We demonstrate that the life-beneficial effect of NMN could be partially reflected by the changes in age-related protein acetylation. Our quantitative assessment indicates that NMN has mild effects on acetylation sites previously reported as substrates of Sirtuins. Collectively, our data analyze protein acetylation with age, laying critical foundation for the functional study of protein post-translational modification essential for healthy aging and perhaps disease conditions.

## Highlights

• Acetylomes from mouse livers are studied with age and NMN treatment.
• Protein acetylation events are discovered as potential aging biomarkers.
• Life-beneficial effect of NMN could be partly reflected by acetylation changes.
• NMN has mild effects on acetylation sites reported as substrates of Sirtuins.

## Graphical Abstract

**Figure:**
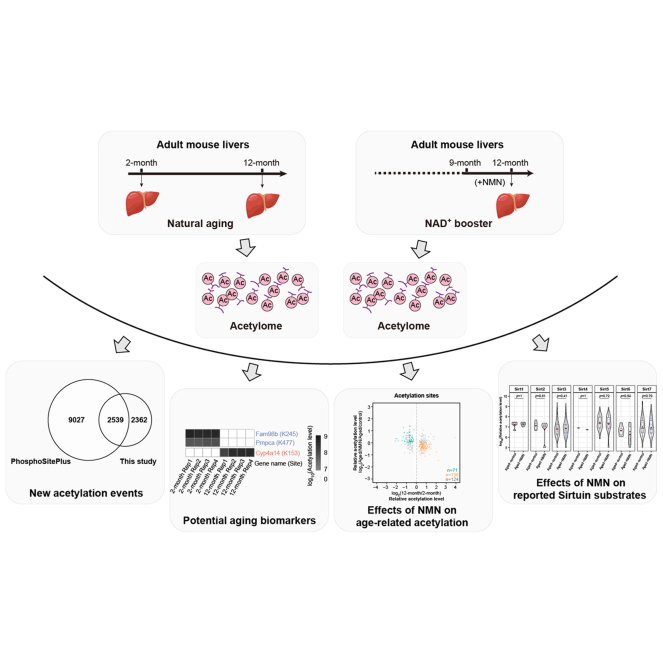

## In Brief

Li *et al*. have performed label-free quantification of the acetylomes in mouse livers with age and upon the treatment of nicotinamide mononucleotide (NMN), revealing previously unknown acetylation events. The datasets characterize not just the dynamics of acetylation with age but also specific acetylation sites as new aging biomarkers. The investigation indicates that the life-beneficial effect of NMN could be partially reflected by the changes in age-related protein acetylation. NMN has mild effects on acetylation previously reported as substrates of Sirtuins.

Protein acetylation is one of the vital protein post-translational modifications (PTMs) that have been shown to impact various cellular processes, including DNA-histone compactness (1), activity of enzymes (2), protein aggregation (3), and protein stability (4). Protein acetylation occurs irreversibly on the α-amino group at the N-terminal amino acid (5) or reversibly on the ε-amino group on the side chain of the lysine residue. Since lysine acetylation is a reversible and thus dynamic process, proteome-wide quantitative characterization of lysine acetylation, the acetylome, is crucial to understand the regulatory role of acetylation. Though proteomic survey of acetylation has been reported in cell lines (6, 7, 8) and organisms, such as fly (9), mouse (10), rat (11), and human (4), dynamics of acetylome during aging process remains poorly described.

Aging is characterized by a progressive loss of physiological integrity, leading to impaired function and increased vulnerability to death (12). Accumulating evidence has indicated critical roles of protein acetylation in aging. For instance, acetylation of Microtubule-associated protein tau (Tau) at K280 has been linked with normal brain aging as well as a wide spectrum of human neurodegenerative diseases including Alzheimer's disease (13, 14), suggesting the potential of lysine acetylation as aging biomarkers. A small-scale acetylome study in the fly head reveals an increase in 79 and a decrease in 3 acetylation sites from 217 high-confidence acetylation events in midlife flies than in those of young flies (9). In mouse hind limb and cardiac muscles, Western blot experiments show that the bulk level of muscle protein acetylation is significantly increased in aged animals (15). However, aging acetylome analysis in mammalian tissues with a resolution at single peptides remains to be addressed.

Nicotinamide adenine dinucleotide (NAD^+^) is a hub metabolite involving in a wide range of cellular processes (16). NAD^+^ has been reported to decline with age in mammalian organs (17, 18, 19). Boosting NAD^+^ biosynthesis, by supplementation of precursory metabolite such as beta-nicotinamide mononucleotide (NMN), triggers life span extension of yeasts (20), worms (21), flies (22), and mice (23), ameliorating age-related diseases (17, 24, 25). Studies have demonstrated that such beneficial effects of NAD^+^ are at least in part due to the activation of Sirtuins (20, 21, 22, 26, 27, 28), a family of NAD^+^-consuming deacetylases (29), indicating changes in acetylome. However, the extent of NAD^+^-induced dynamics in protein acetylation remains unknown. Here, using a dual-antibody enrichment protocol combined with high-resolution liquid chromatography with tandem mass spectrometry (LC-MS/MS) analysis, we performed the label-free quantification of the acetylomes in mouse livers with age and upon the treatment of NMN, revealing previously unknown acetylation events. Our dataset characterized not just the dynamics of acetylation with age but also specific acetylation sites as new aging biomarkers. We further investigated the impact of NMN treatment on protein acetylation.

## Experimental Procedures

### Ethics Statement

All animal procedures have been reviewed and approved by the Institutional Animal Care and Use Committee at the Chinese Academy of Sciences (202003180013) and were in accordance with the Guide for the Care and Use of Laboratory Animals of the Chinese Academy of Sciences. All efforts were made to minimize the suffering of the animals.

### Animal Experimentation

C57BL/6 male mice were housed in an environmentally controlled room under a 12:12-h light/dark cycle at 23 °C and were fed with commercial mouse chow food and water ad libitum. For treatment of beta-nicotinamide mononucleotide (NMN), 9-month-old C57BL/6 mice were supplemented with NMN (Abiochem) dissolved in sterile water (500 mg/kg/d) for 3 months until 12 months of age. NMN water was changed every other day. Four mice were used in 2-month, 12-month and Aged-control experiments, and three mice were used in Aged-NMN experiments. Livers were dissected and immediately frozen in liquid nitrogen.

### Sample Preparation for LC-MS/MS

Mouse liver tissues were homogenized in ice-cold urea lysis buffer containing 8 M urea, 100 mM Tris-hydrochloride (Tris-HCl), pH 8.5, 150 mM sodium chloride (NaCl), and protease inhibitor (Roche). Cell lysates were incubated on ice for 20 min before addition of 1/10 volume 5 M NaCl to release chromatin-bound proteins. The lysates were sonicated to shear genomic DNA and were clarified by centrifugation at 14,000 rcf for 15 min at 4 °C. The protein concentration of the supernatant was determined by the bicinchoninic acid protein assay (Thermo Fisher Scientific). Samples containing 2 mg of proteins were reduced with 5 mM tris(2-carboxyethyl)phosphine (20 min at room temperature), alkylated with 10 mM iodoacetamide (15 min at room temperature in the dark), diluted by a factor of 4 with 100 mM Tris-HCl, pH 8.5 (final concentration of urea was equal to 2 M), and incubated overnight at 37 °C with sequencing-grade trypsin (Promega) added at a 1:100 enzyme:substrate ratio (wt/wt). Samples were then acidified with formic acid (FA) and desalted using solid-phase extraction cartridges C18. Ten micrograms of peptides of each sample was collected as input. The remaining samples were immunoprecipitated with 20 μl of acetyl lysine antibody, agarose (1:1) (ICP0388, ImmuneChem; PTM-104, PTM Biolabs). Agarose was washed by wash buffer I (100 mM NaCl, 1 mM ethylenediaminetetraacetic acid [EDTA], 20 mM Tris-HCl, 0.5% Nonidet P-40; adjust pH to 8.0), wash buffer II (100 mM NaCl, 1 mM EDTA, 20 mM Tris-HCl; adjust pH to 8.0), and ddH_2_O. Finally, the acetylated peptides were eluted with 0.1% trifluoroacetic acid (TFA).

### LC-MS/MS for Proteome and Acetylome

The input peptides and enriched acetylated peptides were analyzed by an online LC-MS system that consisted of an Easy-nLC-1200 HPLC (Thermo Fisher Scientific) and a Q Exactive HF-X mass spectrometer (Thermo Fisher Scientific). Peptides were loaded directly onto a 300-mm-long analytical column (100 μm I.D., 1.9 μm C18 resin) housed in a column oven at 60 °C. A 240-min gradient (3% B at 0 min, 10% B at 2 min, 20% B at 177 min, 40% B at 217 min, 50% B at 224 min, 100% B at 225 and 240 min) (A: 0.1% FA in water, B: 0.1% FA, and 80% acetonitrile [ACN] in water) was developed to separate the peptides at a static flow rate of 300 nl/min. For MS1, the scan range of MS was 350 to 1500 m/z at a resolution of 60,000 and the AGC target was 5e6. The maximum injection time for the precursor ion was 20 ms. Top 10 precursor ions with charge states from 2+ to 6+ were selected for fragmentation. Dynamic exclusion was enabled for 0.5 min. High-energy collisional dissociation was used to fragment the precursor peptides with a normalized collision energy of 27. The resolution of MS2 was set to 60,000, while the AGC target was set to 2e5 with a maximum injection time of 20 ms for input peptides and 120 ms for enriched acetylated peptides.

### Peptide Identification and Quantification

MS data were analyzed by MaxQuant (version 1.6.0.1) (30) and were searched against the SwissProt mouse protein database (released in Jun 2017). The precursor and fragment mass tolerances were 20 ppm. Trypsin was set as the enzyme, and the maximum missed cleavage was set to 2. The carbamidomethylation (+57.0125 Da) of cysteine was set as a static modification. The acetylation (+42.0106 Da) of lysine and protein N-terminal and the oxidation (+15.9959 Da) of methionine were defined as variable modifications. The false discovery rate at peptide spectrum match level was controlled below 1% using a target-decoy approach. “Match between runs” was used during data analysis, and the match time window was set within 0.7 min.

Input (Proteomic) data of mouse livers (2 months and 12 months) were searched together since they were processed in one batch. Input data of NMN-treated and age-matched control mouse livers (Aged-NMN and Aged-control) were analyzed in a separate search. The intensities for protein levels were first divided by the ratio of the sum intensity of each replicate in every group. Then, missing values were labeled as “NA” and the average levels were calculated using intensities in the remaining replicates. Proteins with more than two missing values in each group were filtered out. The ratios of average protein levels of 2 months:12 months and the ratios of average protein levels of Aged-control:Aged-NMN were calculated for normalization of the acetylomic data.

Enrichment (Acetylomic) data of mouse livers (2 months and 12 months) were searched together, and enrichment data of NMN-treated and age-matched control mouse livers (Aged-NMN and Aged-control) were analyzed in another search. The intensities for acetylation levels were first divided by the ratio of the sum intensity of each replicate in every group. Then, missing values were served as “NA” and the average levels were calculated using intensities in the remaining replicates. Acetylation sites with more than two missing values in each group were filtered out. Acetylation sites whose corresponding protein abundances were not quantified were also filtered out. The relative acetylation levels were the acetylation levels divided by the protein ratio of 12-months/2-months or Aged-NMN/Aged-control.

### LC-MS for the Detection of Nicotinamide Adenine Dinucleotide, Oxidized Form (NAD^+^)

Mouse liver tissues were homogenized with 200 μl of H_2_O and 20 ceramic beads (0.1 mm) using the homogenizer (Bertin Technologies, France). Eight hundred microliters of methanol (MeOH):ACN (1:1, v/v) was added for metabolite extraction. To precipitate the protein, samples were incubated for 1 h at −20 °C, followed by 15 min of centrifugation using 14,000 rcf at 4 °C. The supernatants were removed and evaporated to dryness in a vacuum concentrator (Labconco). Dry extracts were then reconstituted in 100 μl of ACN:H_2_O (1:1, v/v), followed by 10 min of sonication (50 Hz, 4 °C) and 15 min of centrifugation using 14,000 rcf at 4 °C to remove insoluble debris. Supernatants were transferred to HPLC glass vials and stored at −80 °C prior to LC-MS analysis.

The LC-MS analysis was performed using a UHPLC system (1290 series, Agilent Technologies) coupled to a triple quadrupole mass spectrometer (Agilent QqQ 6495, Agilent Technologies). A Waters ACQUITY UPLC BEH Amide column (100 × 2.1 mm; particle size, 1.7 μm; 130 Å, Waters) was used for separation, and the column temperature was kept at 25 °C. The mobile phase A was 25 mM ammonium hydroxide (NH_4_OH) and 25 mM ammonium acetate (NH_4_OAc) in water, and B was ACN. The linear gradient was set as follows: 0 to 0.5 min: 95% B, 0.5 to 7 min: 95% B to 65% B, 7 to 8 min: 65% B to 40% B, 8 to 9 min: 40% B, 9 to 9.1 min: 40% B to 95% B, and 9.1 to 12 min: 95% B. The flow rate was 0.5 ml/min, and the sample injection volume was 2 μl. The measurement was performed in positive and negative modes. ESI source parameters were set as follows: sheath gas temperature, 350 °C; dry gas temperature, 170 °C; sheath gas flow, 12 L/min; dry gas flow, 16 L/min; capillary voltage, 3000 V or −2500 V in positive or negative modes, respectively; nozzle voltage, 1000 V and −1500 V in positive and negative modes, respectively; and nebulizer pressure, 40 psi. For the analyses of NAD^+^, several MRM transitions were simultaneously monitored, including 664.1/136.3 for quantification and 664.1/428.0 and 664.1/231.8 for qualification. The dwell time for each MRM transition was 50 ms. The MRM transitions were obtained using the purchased chemical standard of NAD^+^ (10 μg/ml) and optimized using the MassHunter Optimizer software (version B.07.01, Agilent Technologies). The peak areas of quantification transition (*i.e.*, 664.1/136.3) were manually integrated for relative quantitative analysis using the Agilent Qualitative software (version B.07.00, Agilent Technologies).

### Bioinformatic Analysis

Gene Ontology (GO) and Kyoto Encyclopedia of Genes and Genomes (KEGG) pathway analysis were performed by the Database for Annotation, Visualization and Integrated Discovery (DAVID) (version 6.8) (31, 32). Bar plots were generated by GraphPad Prism (version 8.3.0, GraphPad). Venn diagrams were plotted by R package eulerr (version 6.1.1). Correlation matrixes were performed with the ggpairs function of the R package GGally (version 2.1.2). The UpSet plot was generated by R package upSetR (version 1.4.0). Pie chart, scatterplot, and violin plot were produced by R package ggplot2 (version 3.3.5). Heatmaps were plotted by R package pheatmap (version 1.0.12).

## Results

### Acetylome Studies Reveal Previously Uncharacterized Protein Acetylation in Mouse Livers

**Fig. 1:**
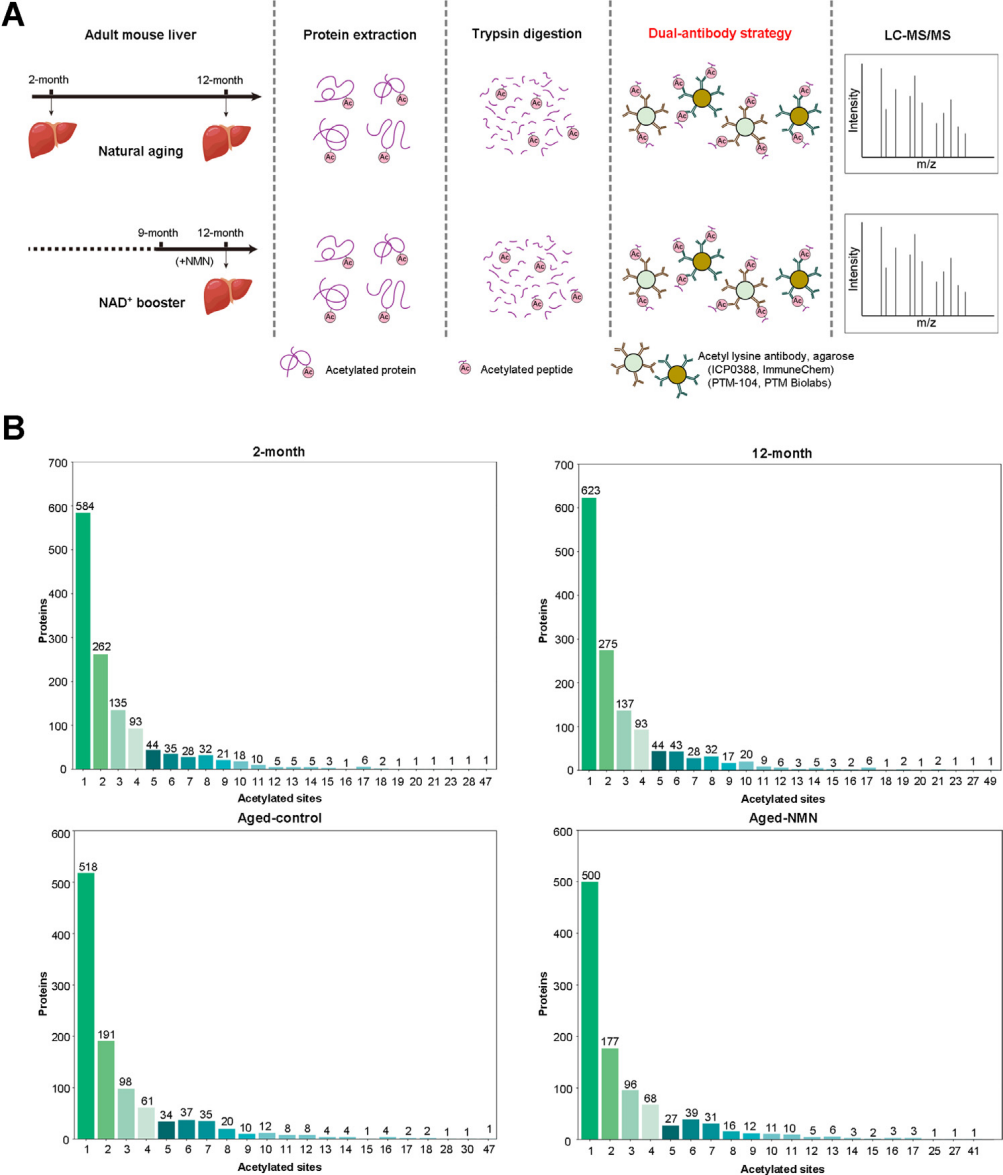
**Global assessment of acetylome in adult mouse livers.***A*, scheme for dual-antibody-based acetylome profiling from adult mouse livers during natural aging and upon the treatment of NAD^+^ booster. *B*, distribution number of the acetylation sites per protein.

To profile protein acetylation with age, we performed proteome-wide acetylation studies. We examined adult mouse at young (2 months) and aged condition (12 months). In addition, we supplemented NMN to a 9-month-old mouse for 3 months to result in 12 months of age (Aged-control *versus* Aged-NMN). To enhance the broadness of epitope coverage for immunoprecipitation, we combined two antibodies for the enrichment of acetylated peptides (Fig. 1*A*). From mouse liver tissues, this strategy led us to identify 4,146, 4,088, 3,251, and 3121 acetylation sites from 2-month-old (four biological replicates), 12-month-old (four biological replicates), Aged-control (four biological replicates), and Aged-NMN (three biological replicates) mice, respectively (supplemental Table S1). Among them, 3,798, 3,915, 2,991, and 2797 acetylation sites were reproducibly identified from at least two out of the biological replicates (supplemental Fig. S1*A* and supplemental Table S1). Comparison of the biological replicates revealed a correlation coefficient equal to or higher than 0.919, indicating high reproducibility (supplemental Fig. S1*B*). Nearly half of acetylated proteins (45.10%, 45.98%, 49.24%, and 49.41% from 2-month-old, 12-month-old, Aged-control, and Aged-NMN mice, respectively) contained one acetylation site, while more than half proteins (54.90%, 54.02%, 50.76%, and 50.59%) had more than one acetylation sites, and notably, a small proportion of proteins (4.71%, 2.07%, 4.56%, and 4.55%) were found to have equal or even higher than 10 acetylation sites (Fig. 1*B*).

**Fig. 2:**
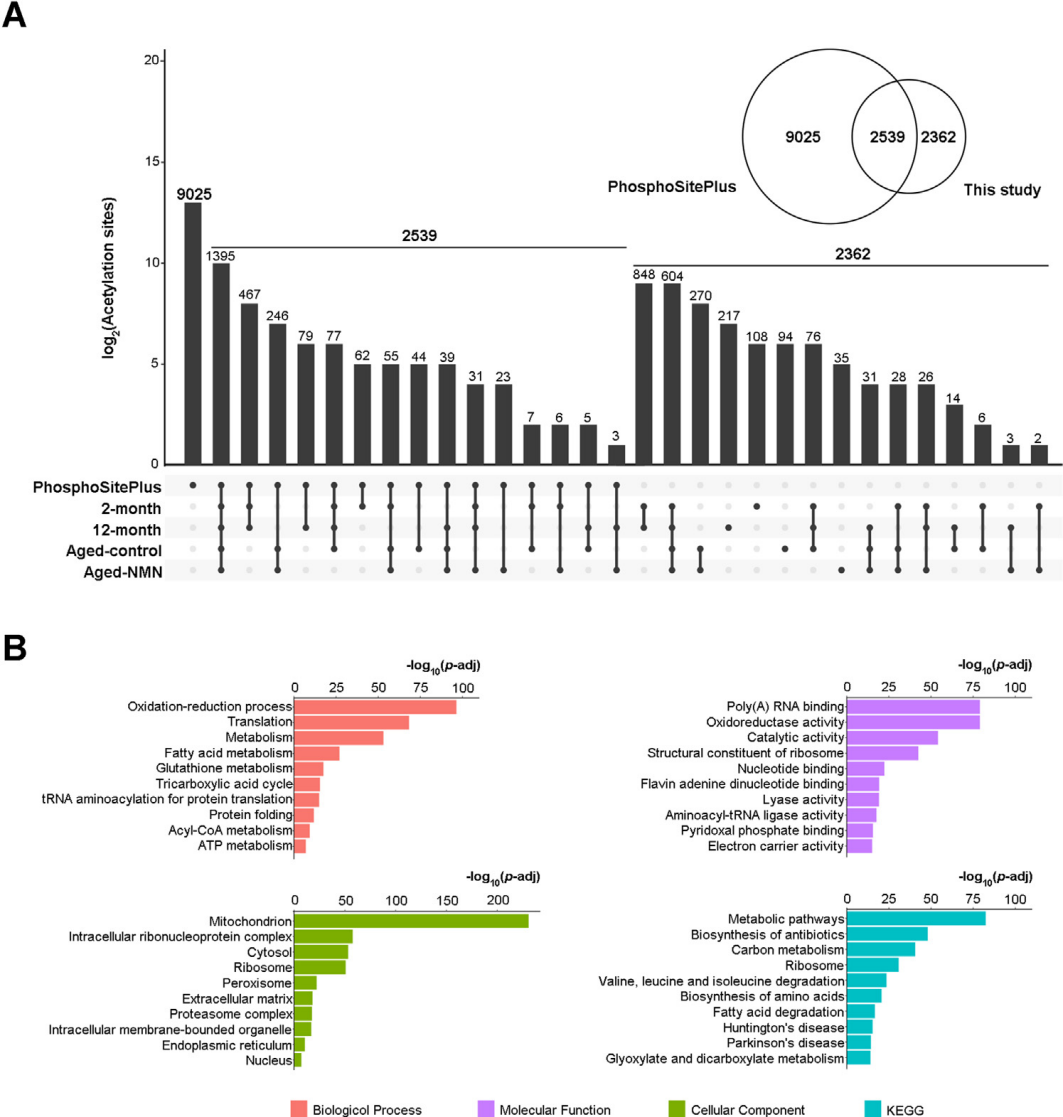
**Acetylome studies reveal previously uncharacterized protein acetylation in mouse livers.***A*, UpSet plot and Venn diagram showing common and specific acetylation sites between PhosphoSitePlus and the current study. *B*, GO analysis reveals biological process, molecular function, cellular component, and KEGG pathway of acetylated proteins in mouse livers.

PhosphoSitePlus comprises 11,564 nonredundant acetylation sites from the mouse (33). Comparative analysis indicated not just 2539 common acetylation sites but also 2362 potentially new acetylation sites as revealed by the current study (Fig. 2*A* and supplemental Table S2), adding new information to the PhosphoSitePlus repertoire. GO on our acetylome data found that acetylated proteins in mouse livers were mainly involved in the oxidation–reduction process, translation, and metabolism (Fig. 2*B* and supplemental Table S2). Molecular functions of the acetylated proteins were primarily in poly(A) RNA binding, oxidoreductase activity, and catalytic activity (Fig. 2*B* and supplemental Table S2). The acetylated proteins were localized in mitochondrion, intracellular ribonucleoprotein complex, and cytosol (Fig. 2*B* and supplemental Table S2). KEGG pathway analysis revealed the enrichment of metabolism pathways, biosynthesis of antibiotics, and carbon metabolism (Fig. 2*B* and supplemental Table S2).

### Age-Associated Dynamics in Protein Acetylation

**Fig. 3:**
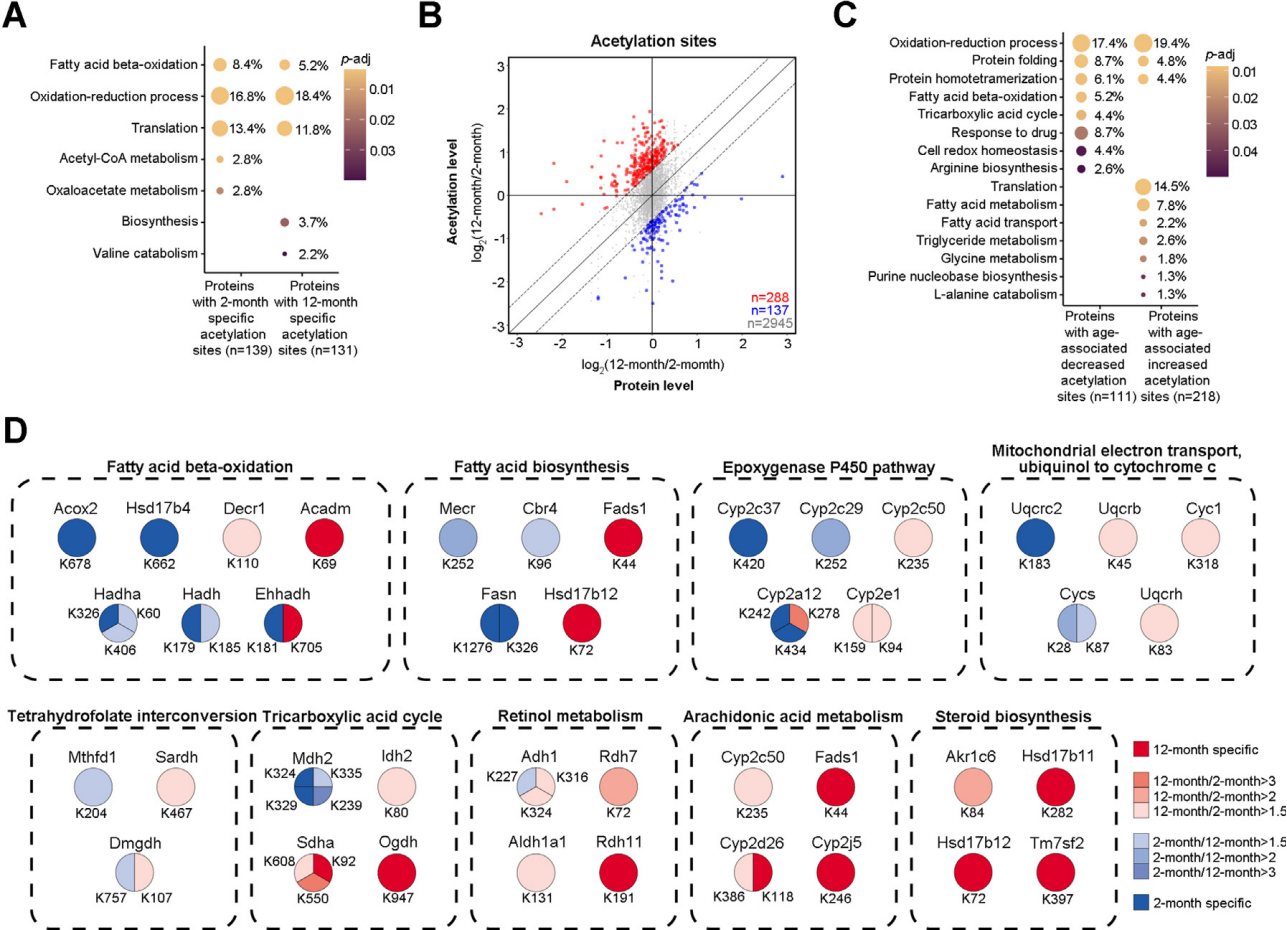
**Age-associated dynamics in acetylation sites.***A*, age-specific acetylated proteins function in diverse biological processes. *B*, scatterplot showing the log2 fold change of 12 months/2 months in acetylation levels as compared to the corresponding protein levels. Each dot on the plot represents a single acetylation site. *Dash lines* indicate the fold change cutoff being 1.5. *Red dots*: age-associated increased sites (12 months/2 months>1.5, *p* < 0.05, two-tailed Student’s *t* test); *blue dots*: age-associated decreased sites (2 months/12 months>1.5, *p* < 0.05, two-tailed Student’s *t* test); *gray dots*: sites with no significant changes. *C*, function of proteins with age-associated decreased or increased acetylation in diverse biological processes. *D*, characterization of acetylated proteins in the oxidation–reduction process with age.

We compared the dynamics of acetylation sites between young and aged animals. To diminish the alternations of protein basal levels that naturally occur with age, changes in acetylation were normalized to the expression of their host protein. Our analysis showed that 155 acetylation sites from 139 proteins were unique in 2-month-old mouse livers (supplemental Fig. S2*A* and supplemental Table S3), with their functions mainly involved in fatty acid beta-oxidation, oxidation–reduction process, translation, acetyl-CoA metabolism, and oxaloacetate metabolism (Fig. 3*A* and supplemental Table S3). On the other hand, 156 acetylation sites from 131 proteins were only identified in 12-month-old mouse livers (supplemental Fig. S2*A* and supplemental Table S3). GO analysis revealed their roles in fatty acid beta-oxidation, oxidation–reduction process, translation, biosynthesis, and valine catabolism (Fig. 3*A* and supplemental Table S3). Among 3370 acetylation sites found in both young and aged animals (supplemental Fig. S2*A* and supplemental Table S3), 137 sites from 111 proteins were significantly decreased with age (2-months/12-months>1.5, *p* < 0.05), while 288 sites from 218 proteins were increased (12-months/2-months>1.5, *p* < 0.05) (Fig. 3*B* and supplemental Table S3). Proteins that had decreased acetylation with age were enriched in oxidation–reduction process, protein folding, protein homotetramerization, fatty acid beta-oxidation, tricarboxylic acid cycle, response to drug, cell redox homeostasis, and arginine biosynthesis (Fig. 3*C* and supplemental Table S3), while acetylation events that had late-onset increase were mainly involved in oxidation–reduction process, translation, protein folding, protein homotetramerization, metabolic processes of fatty acid, triglyceride, and glycine (Fig. 3*C* and supplemental Table S3). During aging, acetylation events involved in the oxidation–reduction process were notably enriched (Fig. 3, *A* and *C*). Detailed characterization determined that acetylation of the redox proteins in tricarboxylic acid cycle was upregulated with age except for Malate dehydrogenase, mitochondrial (Fig. 3*D* and supplemental Table S3). Apart from K227 on Alcohol dehydrogenase 1, protein acetylation in retinol metabolism increased with age (Fig. 3*D* and supplemental Table S3). Proteins in arachidonic acid metabolism and steroid biosynthesis demonstrated increased acetylation with age (Fig. 3*D* and supplemental Table S3).

**Fig. 4:**
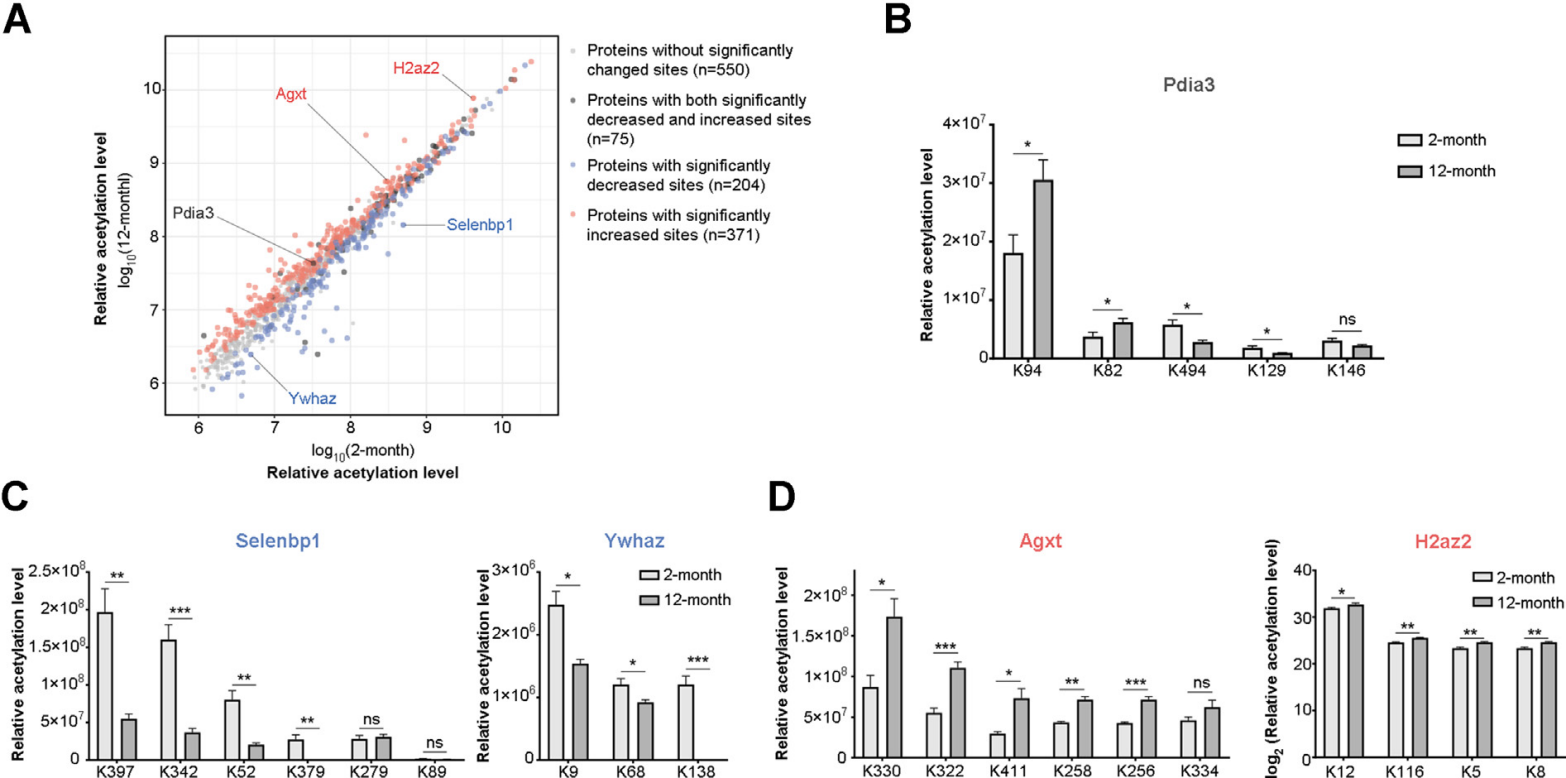
**Age-associated dynamics in acetylated proteins.***A*, scatterplot showing distribution of the sum acetylation levels of all identified sites on individual proteins (12 months vs. 2 months). Each dot represents a single acetylated protein. *Light gray dots*: proteins without significantly changed sites; *dark gray dots*: proteins with both significantly decreased and increased sites (*p* < 0.05 by two-tailed Student’s *t* test); *blue dots*: proteins with significantly decreased acetylation sites (*p* < 0.05, two-tailed Student’s *t* test); *red dots*: proteins with significantly increased acetylation sites (*p* < 0.05, two-tailed Student’s *t* test). *B*, bar plots showing relative acetylation levels of representative protein with discordant dynamics. *C*, bar plots showing proteins with uniformly decreased acetylation sites. *D*, bar plots showing proteins with consistently increased acetylation sites with age (two-tailed Student’s *t* test: ∗∗∗*p* < 0.001; ∗∗*p* < 0.01; ∗*p* < 0.05; ns, not significant).

We calculated the sum acetylation levels of all identified sites on each protein and counted the numbers of dramatically changed acetylation sites on each protein (*p* < 0.05) (Fig. 4*A* and supplemental Table S4). From a total number of 1200 proteins identified from 2-month-old and 12-month-old mouse livers, acetylation in 550 proteins remained roughly unaltered. Interestingly, 75 proteins had multiple acetylation events conferring discordant dynamics with age, with some being increased while others being decreased (*e.g.*, Protein disulfide-isomerase A3) (Fig. 4*B* and supplemental Table S4); 204 proteins had uniformly decreased acetylation sites (*e.g.*, Methanethiol oxidase and 14-3-3 protein zeta/delta) (Fig. 4*C* and supplemental Table S4), and acetylation in 371 proteins became significantly increased in aged animals (*e.g.*, Mitochondrial serine-pyruvate aminotransferase and Histone H2A.V) (Fig. 4*D* and supplemental Table S4).

### Protein Acetylation as New Age-Associated Biomarkers

**Fig. 5:**
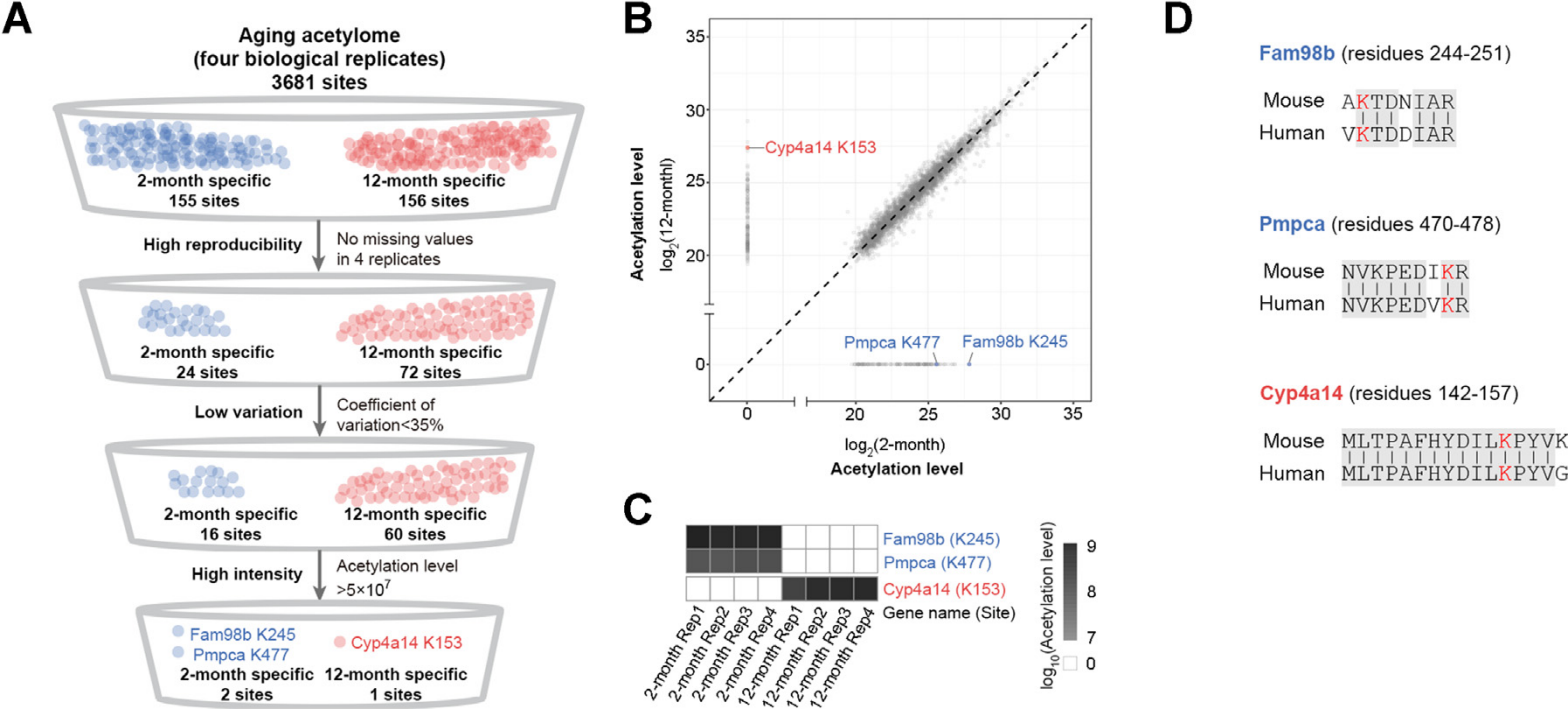
**Protein acetylation as new age-associated biomarkers.***A*, scheme for filtering age-associated biomarkers. *B*, scatterplot showing the log2 intensity of acetylation levels in 12 months as compared to 2 months. Each dot represents a single acetylation site. *Blue dots*: biomarkers for young mouse livers; *red dot*: biomarker for aged mouse livers. *C*, heat map showing the acetylation levels of age-associated biomarkers. *D*, sequence alignment analysis reveals the evolutionarily conservation of biomarkers between mouse and human.

To explore protein acetylation as biomarkers, we designed a scheme to filter acetylation sites based on specificity with age, high reproducibility, low variation among repeats, and high intensity (Fig. 5*A*). High reproducibility was defined by acetylation signals detected from all four replicates for each age group. Low variation was defined by acetylation signals with coefficient of variation below 35% among replicates. High intensity referred to the average acetylation levels higher than 5 × 10^7^. This scheme led to us to identify two young-specific (2-months) acetylation sites (Protein FAM98B at K245 and Mitochondrial-processing peptidase subunit alpha at K477) and one age-associated (12-months) acetylation sites (Cytochrome P450 4A14 at K153) (Fig. 5, *A* and *B*). Protein FAM98B at K245 and Mitochondrial-processing peptidase subunit alpha at K477 were highly acetylated in 2-month-old mouse livers but were barely detectable at 12 months of age (Fig. 5*C*). On the contrary, the acetylation of Cytochrome P450 4A14 at K153 was not identified in 2-month-old animals but was found highly modified in age mouse livers (Fig. 5*C*). Sequence alignment analysis revealed that these lysine sites were evolutionarily conservative between mouse and human, suggesting their potential as aging biomarkers in mammals (Fig. 5*D*).

### Impact of NMN Treatment on Protein Acetylation

**Fig. 6:**
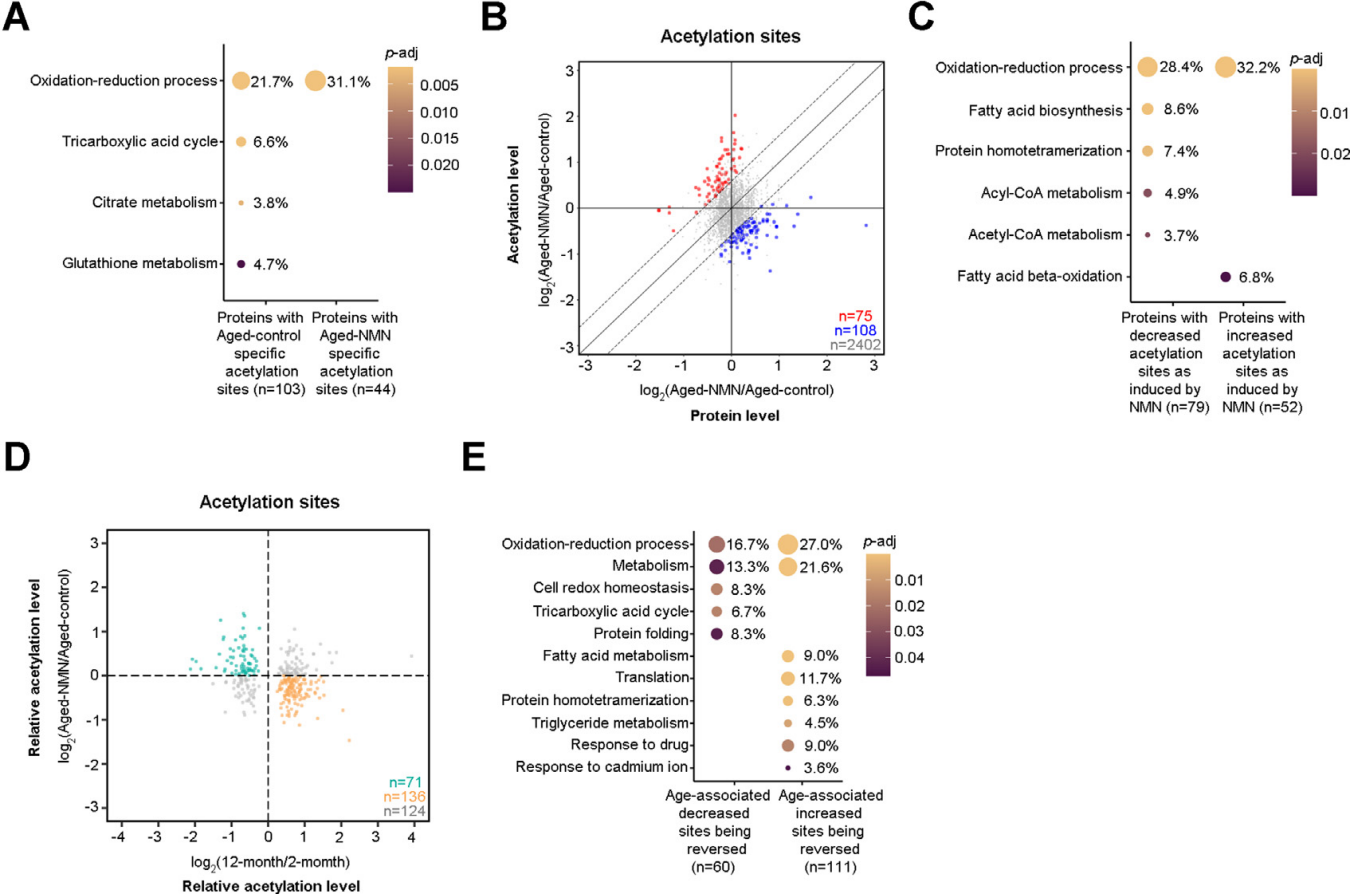
**Impact of NMN treatment on protein acetylation.***A*, NMN-induced acetylated proteins function in diverse biological processes. *B*, scatterplot showing the log2 fold change of Aged-NMN/Aged-control in acetylation levels as compared to their host protein levels. Each dot represents a single acetylation site. *Dash lines* indicate the fold change cutoff being 1.5. *Red dots*: NMN-induced increased sites (Aged-NMN/Aged-control>1.5, *p* < 0.05, two-tailed Student’s *t* test); *blue dots*: NMN-induced decreased sites (Aged-control/Aged-NMN>1.5, *p* < 0.05, two-tailed Student’s *t* test); *gray dots*: sites with no significant changes. *C*, function of proteins with decreased or increased acetylation as induced by NMN in diverse biological processes. *D*, scatterplot showing age-associated decreased sites (*p* < 0.05, two-tailed Student’s *t* test) being reversed by NMN (*green dots*) and age-associated increased sites (*p* < 0.05, two-tailed Student’s *t* test) being reversed by NMN (*yellow dots*). *E*, NMN-reversed acetylated proteins function in diverse biological processes.

We characterized the effect of NMN supplement on the acetylome of aged animals. Changes in acetylation were also normalized to the host protein. One hundred fifteen acetylation sites from 103 proteins were specifically identified in Aged-control mouse livers (supplemental Fig. S2*B* and supplemental Table S5). GO analysis showed that they were enriched in oxidation–reduction process, tricarboxylic acid cycle, citrate metabolism, and glutathione metabolism (Fig. 6*A* and supplemental Table S5). Only 46 acetylation sites from 44 proteins were uniquely identified in the Aged-NMN group (supplemental Fig. S2*B* and supplemental Table S5), with their roles in the oxidation–reduction process (Fig. 6*A* and supplemental Table S5). Among 2585 acetylation sites identified in both Aged-control and Aged-NMN animals (supplemental Fig. S2*B* and supplemental Table S5), 108 sites from 79 proteins were significantly decreased upon the treatment of NMN (Aged-control/Aged-NMN>1.5 and *p* < 0.05) (Fig. 6*B* and supplemental Table S5); these proteins were enriched in oxidation–reduction process, fatty acid biosynthesis, protein homotetramerization, acyl-CoA metabolism, acetyl-CoA metabolism, and fatty acid beta-oxidation (Fig. 6*C* and supplemental Table S5). On the other hand, 75 sites from 52 proteins had increased acetylation (Aged-NMN/Aged-control>1.5 and *p* < 0.05) (Fig. 6*B* and supplemental Table S5), with their roles being enriched in oxidation–reduction process and fatty acid beta-oxidation (Fig. 6*C* and supplemental Table S5).

Since NMN supplementation has been shown to benefit health span (17, 20, 21, 22, 23, 24), we further investigated the impact of NMN on protein acetylation that normally changed during aging. Seventy-one acetylation sites in 60 proteins that naturally decreased with age were now reversed by NMN treatment (Fig. 6*D* and supplemental Table S5). GO analysis revealed their roles in oxidation–reduction process, metabolism, cell redox homeostasis, tricarboxylic acid cycle, and protein folding (Figs. 6*E*, S3*A* and supplemental Table S5). One hundred thirty-six acetylation sites in 111 proteins that had age-modulated increase became decreased with NMN (Fig. 6*D* and supplemental Table S5), which were mainly involved in oxidation–reduction process, metabolic processes of fatty acid and triglyceride, translation, protein homotetramerization, response to drug, and response to cadmium ion (Figs. 6*E*, S3*A* and supplemental Table S5). In addition, 17 2-month-specific acetylation sites and 17 12-month-specific acetylation sites were reversed by NMN (supplemental Fig. S3*B* and supplemental Table S5). We showed the evidence that 3 acetylation sites that were highly enriched in young animals now became abundant in NMN-treated mouse livers compared to age-matched controls (supplemental Fig. S3*B* and supplemental Table S5). In addition, 14 acetylation sites found be high in aged animals were significantly decreased in NMN-treated mouse livers (supplemental Fig. S3*B* and supplemental Table S5). Notably, the acetylation level of the aging biomarker Cytochrome P450 4A14 at K153 (see Fig. 5*C*) was downregulated upon NMN treatment compared to the age-matched controls (supplemental Fig. S3*B*). The acetylation of Protein FAM98B at K245 and Mitochondrial-processing peptidase subunit alpha at K477 as young biomarkers were not detectable in NMN-treated animals (12 months).

### NMN Treatment Mildly Affects Sirtuin-Mediated Protein de-Acetylation

**Fig. 7:**
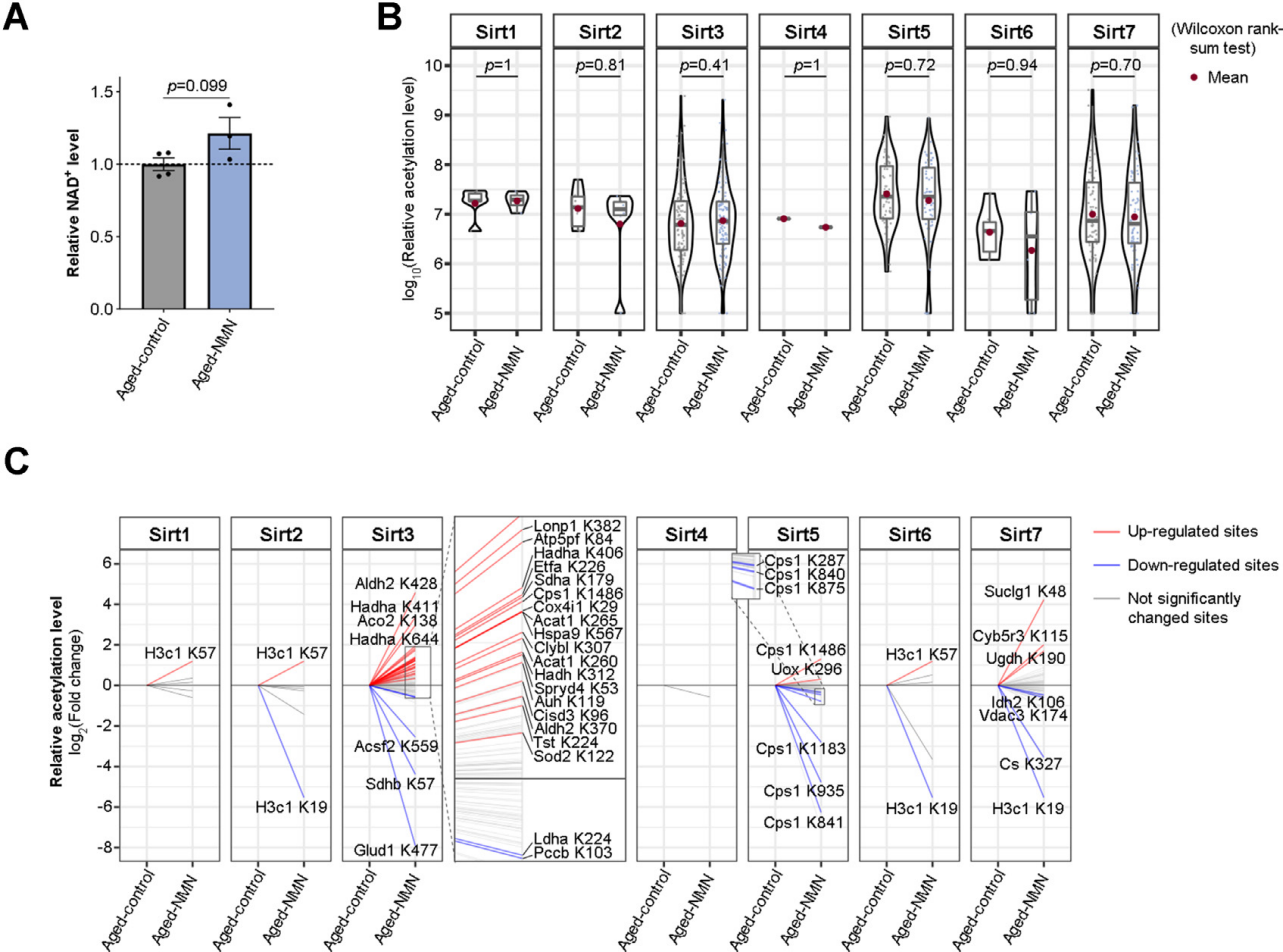
**NMN treatment mildly affects Sirtuin-mediated protein deacetylation.***A*, NMN supplementation causes an increase in NAD^+^ level. *B*, violin plot showing global dynamics of the acetylation levels of Sirtuin substrates by NMN treatment (Wilcox rank-sum test). *C*, *line chart* showing the dynamics of the acetylation levels of Sirtuin substrates by NMN treatment. *Red lines*: NMN-induced upregulated sites (*p* < 0.05 by two-tailed Student’s *t* test); *blue lines*: NMN-induced downregulated sites (*p* < 0.05 by two-tailed Student’s *t* test); *gray lines*: not significantly changed sites.

It has been suggested that NMN supplementation, coupled with an increased NAD^+^ level (Fig. 7*A*), benefits adult fitness at least in part by promoting the activity of Sirtuins, a family of NAD-consuming deacetylases (20, 21, 22, 26, 27, 28). In our Aged-control and Aged-NMN dataset, we collectively identified 250 acetylation sites previously reported as substrates of Sirtuins (34, 35, 36, 37, 38, 39, 40, 41, 42, 43, 44, 45, 46, 47, 48, 49, 50, 51, 52, 53, 54, 55, 56, 57, 58, 59, 60, 61, 62, 63) (supplemental Table S6). Surprisingly, overall levels of the acetylation sites remained roughly unchanged upon the treatment of NMN compared to those of age-matched controls (Fig. 7*B* and supplemental Table S6). We only observed 42 out of 250 acetylation sites altered by NMN supplementation, with 15 sites displaying decreased modification, while 27 sites being increased (Fig. 7*C* and supplemental Table S6). Changes in acetylation may impact protein function, yet there have been functional studies for 3 out of 42 acetylation sites being affected. Specifically, Histone H3.1 at K19 (H3K18Ac after N-terminal methionine excision co-translationally by the enzyme Methionine aminopeptidase 2) (64) had decreased acetylation upon NMN treatment (Fig. 7*C*), while acetylation on Histone H3.1 at K57 (H3K56Ac), with a role in transcriptional activation (65), was increased (Fig. 7*C*). NMN treatment also increased the acetylation of Sirt3 substrate Superoxide dismutase [Mn], mitochondrial at K122 (Fig. 7*C*), which has been reported to decrease the enzymatic activity as destroying superoxide anion radicals produced within the cells (66).

## Discussion

Aging is characterized by a progressive decline in cellular and organismal function that leads to reduction of fitness and increased risks to diseases and death. Aging is a complex process that can be regulated by a network of multiple mechanisms. Previously studies have focused on age-related changes on transcriptomics (67, 68), epigenetics (69), proteomics (70), and metabolomics (71), while research on protein PTM, such as acetylation, remains elusive. To investigate the dynamics of acetylation with age and upon the treatment of NMN, a precursor of health-promoting metabolite NAD^+^, we performed dual-antibody enrichment experiments combined with high-resolution LC-MS/MS analysis in mouse livers. To our knowledge, our study provides the first comprehensive and dynamic atlas of acetylome in natural aging and upon treatment of NAD^+^ booster from adult mouse tissues.

Protein lysine acetylation has been linked with cellular functions including cell cycle (72), DNA damage repair (73), autophagy (74), and diseases such as diabetes (75) and cancer (76). Our quantitative assessment reveals new features of aging acetylome from mouse livers. First, we identify 4901 acetylation sites in mouse livers, with 48.19% (2362) being previously unknown acetylation sites in PhosphoSitePlus. Second, 7.93% sites (292 out of 3681) are decreased with age, while 12.06% sites (444 out of 3681) show increased acetylation in aged animals. We observe more acetylation sites in aged mice than in young animals. Third, proteins with a similar function may have differential acetylation with age. For example, within the biological process of oxidation–reduction, Fatty acid synthase at K326 and K1276 show decreased acetylation, while Cytochrome P450 2E1 at K94 and K159 are increased with age. Fourth, 58.83% proteins (706 out of 1200) bear more than one acetylation modification. Interestingly, 6.25% of proteins (75 out of 1200) have multiple acetylation events conferring discordant direction of change during aging. For example, the acetylation of Peroxisomal bifunctional enzyme at K181 displays a decrease, while K705 has an increase with age. Individual proteins might be substrates of different lysine acetyltransferases and/or lysine deacetylases. For example, Histone H3 is acetylated by Gcn5 and PCAF at K9 (77) and acetylated by p300/CBP at K18, K27 (78), and K56 (79). Similarly, single-lysine sites could be modulated by different enzymes, *e.g.*, Histone H3 at K56 is deacetylated by Sirt1, Sirt2, and Sirt6 (79, 80, 81). This might partially explain why some proteins harbor both increased and decreased acetylation sites. However, how multiple acetylation sites coordinate to affect protein function has not been studied. Our datasets provide candidates for the exploration of joint outcome by multisite acetylation on individual proteins.

During aging, acetylation on proteins in the oxidation–reduction process showed complex changes (see Fig. 3*D*). The oxidation–reduction process (GO: 0055114) is a metabolic process that mediates the removal or addition of one or more electrons to or from a substance, with or without the concomitant removal or addition of a proton or protons. Altered cellular redox homeostasis has been implicated as an important factor in regulating cell growth, senescence, and aging (82). The liver is a critical tissue for numerous metabolic processes including digestive absorption and uptake, synthesis, packaging, and secretion of lipids and lipoproteins (83). Here, we observed dynamic acetylation on proteins involved in fatty acid metabolism (including increased and decreased acetylation in fatty acid beta-oxidation and increased acetylation in fatty acid transport) and increased acetylation on proteins in triglyceride metabolism in aging mouse livers. The liver is also important for protein and amino acid metabolism as it is responsible for most proteins secreted into the blood (83). We found that proteins in valine catabolism, glycine metabolism, and L-alanine catabolism showed increased acetylation, which may potentially underlie functional changes in liver tissues that occur during aging.

It is interesting to note that Peleg *et al.* (9) have performed acetylomic study in *Drosophila*, revealing an increase in 79 and a decrease in 3 acetylation sites in midlife flies compared to young flies. We have performed comparative analysis between their and our datasets. ADP/ATP translocase (sesB) at K107 shows an increase in *Drosophila* head during aging, and its mouse ortholog ADP/ATP translocase 1 (Slc25a4) at K92 also increases in old mouse livers. The acetylation levels of *Drosophila* Aspartate aminotransferase (Got2) at K310 and mouse Aspartate aminotransferase, mitochondrial (Got2) at K309 both increase during aging. This evidence highlights conserved acetylation events that occur with age.

Protein acetylation may be used as aging biomarkers. It has been shown that the acetylation of K280 on Tau is linked with normal brain aging as well as a wide spectrum of human neurodegenerative diseases (13, 14). Here, we extend the scope by exploring aging acetylome, revealing new protein acetylation sites as age-related biomarkers. Specifically, we demonstrate the acetylation of Protein FAM98B at K245 and Mitochondrial-processing peptidase subunit alpha at K477 as biomarkers for young mouse livers and Cytochrome P450 4A14 at K153 as biomarker for aged animals.

The hub metabolite, NAD^+^, influences cellular physiology though multiple dimensions. Studies have shown that NAD^+^ modulates the cellular redox status (16), modifies mRNA by capping the transcript at the 5′-end (84), and rewires the acetylome by activating deacetylases, Sirtuins (20, 21, 22, 26, 27, 28). However, the extent by which NAD^+^-induced changes in protein acetylation remains unknown. By assessing protein acetylation that normally changes with age, we note that 60.14% acetylation sites (258 out of 429) can be reversed by NMN treatment, including Cytochrome P450 4A14 at K153, the aging biomarker for aged mouse livers, suggesting that the life-beneficial effect of NMN can be at least partially reflected by changes in age-related protein acetylation. We further provide the evidence to show that NMN elicits a decrease in acetylation sites. It is still unknown whether such a decrease is due to Sirtuin-mediated deacetylation. In our dataset, however, majority of acetylation sites previously reported as substrates of Sirtuins show mild or even no change, with only 6% acetylation sites (15 out of 250 reported substrates of Sirtuins) being decreased by NMN supplementation. Though it remains to be determined, it is possible that unknown target sites may be responsive to NAD^+^-activated Sirtuins. Alternatively, nicotinamide (NAM), a metabolite converted from NAD^+^ during Sirtuin-mediated deacetylation (85), has been shown to inhibit the enzymatic activity of Sirtuins (86, 87). As a consequence, despite the fact that NAD^+^ activates Sirtuins, accumulation of NAM leads to the inhibition of Sirtuins, resulting in a perplexed outcome in protein deacetylation status. We also notice that Sirt3 appears to have primarily upregulated sites while Sirt5 has downregulated sites upon the treatment of NMN (see Fig. 7*C*). Sirt3 as well as Sirt5 are mainly localized in the mitochondrial matrix (81, 88), competing with the same NAD^+^ pool. Previous studies have indicated that NAD^+^ consuming Michaelis constant (Km) value for Sirt5 (ranging from 26 μM (89) to 200 μM (90)) is lower than that of Sirt3 (ranging from 280 μM (91) to 880 μM (92)). It is tempting to speculate that Sirt5 can be activated prior to Sirt3. Activation of Sirt5 results in a rapid depletion of NAD^+^ and an accumulation of NAM (86, 87), which, in turn, leads to inhibition of Sirt3-mediated deacetylation.

In summary, our study provides an important resource on protein lysine acetylation that occurs with age, laying critical foundation for the functional study of protein PTM essential for healthy aging and perhaps disease conditions.

## Data Availability

The MS data have been deposited under the accession number IPX0004109000 at iProX (93) with the dataset identifier PXD031851. (https://www.iprox.cn/page/project.html?id=IPX0004109000)

## Supplemental data

This article contains supplemental data (34, 35, 36, 37, 38, 39, 40, 41, 42, 43, 44, 45, 46, 47, 48, 49, 50, 51, 52, 53, 54, 55, 56, 57, 58, 59, 60, 61, 62, 63).

## Conflict of interest

The authors declare that they have no conflicts of interest with the contents of this article.

## Figures and Tables

**Figure S1:**
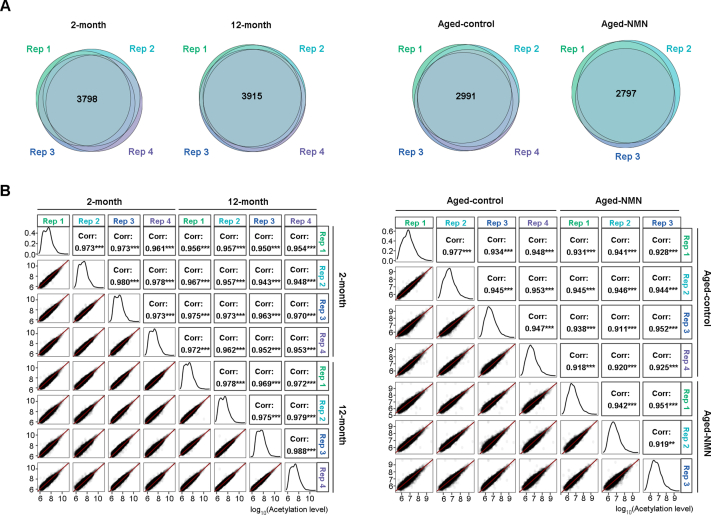


**Figure S1:**
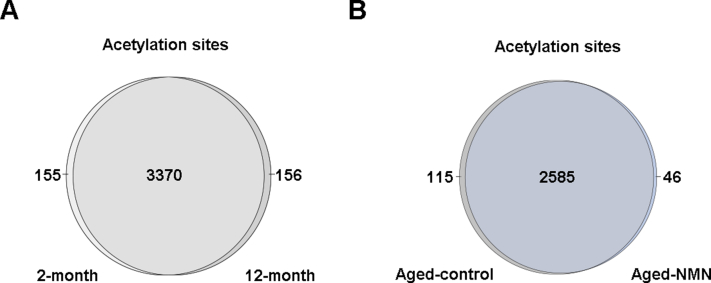


**Figure S1:**